# Home or hospital for people with dementia and one or more other multimorbidities: What is the potential to reduce avoidable emergency admissions? The HOMEWARD Project Protocol

**DOI:** 10.1136/bmjopen-2017-016651

**Published:** 2017-04-03

**Authors:** S Voss, S Black, J Brandling, M Buswell, R Cheston, S Cullum, K Kirby, S Purdy, C Solway, H Taylor, J Benger

**Affiliations:** 1 Faculty of Health and Applied Sciences, University of the West of England, Bristol, UK; 2 Research and Development Department, South Western Ambulance Service NHS Foundation Trust, Exeter, UK; 3 Centre for Research in Primary and Community Care, University of Hertfordshire, Hatfield, UK; 4 Faculty of Medical and Health Sciences, University of Auckland, Auckland, New Zealand; 5 School of Social and Community Medicine, University of Bristol, Bristol, UK; 6 Alzheimer's Society Research Network, London, UK; 7 Research Design Service South West, University Hospitals Bristol NHS Foundation Trust, Bristol, UK

**Keywords:** ACCIDENT & EMERGENCY MEDICINE

## Abstract

**Introduction:**

Older people with multimorbidities frequently access 999 ambulance services. When multimorbidities include dementia, the risk of ambulance use, accident and emergency (A&E) attendance and hospital admission are all increased, even when a condition is treatable in the community. People with dementia tend to do poorly in the acute hospital setting and hospital admission can result in adverse outcomes. This study aims to provide an evidence-based understanding of how older people living with dementia and other multimorbidities are using emergency ambulance services. It will also provide evidence of how paramedics make decisions about taking this group of patients to hospital, and what resources would allow them to make more person-focused decisions to enable optimal patient care.

**Methods and analysis:**

*Phase 1: retrospective data analysis*: quantitative analysis of ambulance service data will investigate: how often paramedics are called to older people with dementia; the amount of time paramedics spend on scene and the frequency with which these patients are transported to hospital. *Phase 2: observational case studies*: detailed case studies will be compiled using qualitative methods, including non-participant observation of paramedic decision-making, to understand why older people with multimorbidities including dementia are conveyed to A&E when they could be treated at home or in the community. *Phase 3: needs analysis*: nominal groups with paramedics will investigate and prioritise the resources that would allow emergency, urgent and out of hours care to be effectively delivered to these patients at home or in a community setting.

**Ethics and dissemination:**

Approval for the study has been obtained from the Health Research Authority (HRA) with National Health Service (NHS) Research Ethics Committee approval for phase 2 (16/NW/0803). The dissemination strategy will include publishing findings in appropriate journals, at conferences and in newsletters. We will pay particular attention to dissemination to the public, dementia organisations and ambulance services.

**Strengths and limitations of this study**

- Findings from the research will inform service-level developments to reduce avoidable hospital admission for older people.
- This study will quantify the amount of time ambulance staff spend on the scene when called to people with dementia and other multimorbidities.
- The use of non-participant observation will allow the investigation of complex and interacting factors that influence paramedic decision-making.
- Analysis of ambulance data is retrospective; thus, there is the potential for selection bias in defining the control and target groups.
- Paramedics participating in case studies are a self-selecting sample, which limits the generalisability of the study findings.

## Introduction

### Background

Dementia is a progressive and irreversible condition, which can have devastating consequences for patients and families. As the disease advances, cognitive, functional, behavioural and psychological abilities decline with an associated loss of independent living and social interaction. The number of people living with dementia is steadily increasing. In 2013, there were an estimated 815 827 people with dementia in the UK; 773 502 were aged 65 years or over. This represents 1 in every 14 of the population aged 65 years and over.^1^ The steadily increasing demands on urgent and emergency healthcare services are well documented and widespread. There are ∼8 million 999 ambulance calls and 20 million accident and emergency (A&E) attendances in the UK every year. While the majority of urgent care is delivered in primary care settings, an increasing number of older people are accessing ambulance services and A&E departments.

People with dementia are dealing with, on average, 2–8 additional chronic diseases (multimorbidities)^2^ ^3^ and dementia is associated with higher levels of multimorbidity than in an age-matched population.^4^ ^5^ Unsurprisingly then, dementia is associated with an increased risk of hospitalisation.^6^ Notably, when multimorbidities are adjusted for, people with dementia have a higher incidence of A&E attendance^4^ and are more likely to access the ambulance service.^6^ This means that where a patient's multimorbidities include dementia, the risk of ambulance use, A&E attendance and hospital admission are all increased, compared with patients with the same multimorbidities not including dementia. The ‘conversion rate’ (proportion of A&E attendances arriving by ambulance that become a hospital admission) is very high in this patient group;^7^ therefore, the vast majority of older people with multimorbidities including dementia transported to A&E are then admitted to hospital, often unnecessarily. This is particularly important as patients with dementia tend to do poorly in ambulance and acute hospital settings; cognitive impairment results in a reduced threshold for sensory overload and distress which can lead to disruptive behaviours.^8–10^

Furthermore, dementia can be a significant barrier for ambulance staff and clinicians in A&E; confusion resulting from dementia may contribute to inaccuracies in the medical or medication history, difficulties gathering a history of the present illness, and an individual's ability to comprehend or follow discharge instructions. The concept of ‘diagnostic overshadowing’ originates from learning disabilities research, but can also be applied to mental health and dementia.^11–13^ Diagnostic overshadowing occurs when multimorbid conditions mask the symptoms of dementia or conversely, there is the possibility that dementia could mask the existence of problematic multimorbid conditions. The combination and interaction of these factors can lead to adverse clinical outcomes,^14^ and if admitted for inpatient hospital care, older people with multimorbidities including dementia have the highest readmission rates and highest rate of long-term care use after discharge.^15^ ^16^

The vast majority of changes in long-term health conditions such as heart failure, chronic obstructive pulmonary disease and diabetes do not require hospital treatment. Minor deteriorations, exacerbations or complications can all be dealt with at home or in the community providing effective and timely access to responsive health and care services that exist. However, where dementia is a multimorbidity, the risk of 999 ambulance attendance, A&E attendance and acute hospital admission are all increased, despite the fact that these are potentially avoidable and lead to poor outcomes and experiences for patients and carers. Reducing avoidable conveyance to A&E and acute hospital admission for older people with multimorbidities including dementia would be beneficial to patients and the National Health Service (NHS).

### Rationale

Older people with multimorbidities including dementia may call emergency services on 999 in a confused state and with a real or perceived need to obtain care and urgent support. For paramedics, these situations present multiple challenges. In the absence of a reliable carer or informant, they may be unable to obtain an accurate history. If the call is out of hours, they are often unable to contact other services for advice or to obtain community follow-up. In these cases, paramedics have little choice but to convey the patient to A&E for assessment, even knowing that this course of action is likely to be inappropriate and potentially detrimental to the patient, their family and the wider health service.

The use of emergency medical services (EMS) by older people with multimorbidities including dementia is not well understood, and a review by Buswell *et al*^17^ highlights the issue of ‘inappropriate’ calls where an ambulance is the last resort or a ‘safety net’. The authors identified recurrent themes including the absence of an alternative to hospital, a lack of integration in healthcare and that 999 is the default option. Although few of these assumptions have been tested empirically, there is an emerging consensus about the lack of information, information sharing, service integration and missed opportunities for ambulance crews when attending people at home. However, these issues have not been studied in older people with multimorbidities including dementia, who have additional health and care needs. This is particularly true for situations where multimorbidities would not in themselves lead to ambulance use and hospital admission, but when combined with dementia are associated with an increased and prolonged use of emergency and acute hospital services that could potentially be avoided.

### Aims and objectives

This study will provide an evidence-based understanding of how older people living with dementia and other multimorbidities are using emergency ambulance services. It will also provide evidence of how paramedics make decisions about taking this group of patients to hospital, and what resources would allow them to make more person-focused decisions to enable optimal patient care.

The overarching aim of this project is to explore the potential to reduce avoidable emergency admissions for older people with multimorbidities including dementia. This is particularly important because older people with dementia often do poorly in the acute hospital setting; hospital admission can result in adverse clinical outcomes. The objectives are to: assess the burden that older people with multimorbidities including dementia place on the ambulance service; investigate factors influencing paramedic decision-making when managing these patients; and explore potential alternatives that could avoid unnecessary A&E attendances and hospital admissions.

The outputs will be fundamental to further planned research initiatives which will develop and evaluate interventions that allow emergency, urgent and out of hours care to be effectively delivered at home or in a community setting.

### Research question

What factors influence the decision to transport an older person with multimorbidities, including dementia, to hospital when they access the ambulance service with a problem that could be managed at home or in the community?

This question will be addressed in three phases of work.

### Phase 1: retrospective data analysis

Quantitative analysis of ambulance service data will be used to investigate:

- How often dementia and other multimorbidities are recorded;
- The number and proportion of older people with multimorbidities including dementia that are transported to hospital, in comparison to control calls;
- The reasons given for transporting older people with multimorbidities including dementia to hospital;
- How long calls to people with multimorbidities including dementia last, in comparison to control calls.

### Phase 2: observational case studies

We will use qualitative methods, including non-participant observation, to understand why older people with multimorbidities including dementia are conveyed to A&E when they could be treated at home or in the community. Detailed case studies will be compiled using a combination of observation, interview and documentation analysis, to study the factors influencing the paramedic decision-making process.

### Phase 3: needs analysis

We will use nominal group meetings to identify what resources should be available to paramedics to allow emergency, urgent and out of hours care to be effectively delivered to older people with multimorbidities including dementia at home or in a community setting, rather than conveyance to A&E by ambulance and the resulting risk of hospital admission.

## Methods and analysis

### Phase 1: retrospective ambulance service data analysis

Ambulance patient care records (PCRs) from calls to patients aged 65 years and over in the geographical areas covered by South Western Ambulance Service NHS Trust (SWASFT) West Division and East of England Ambulance Service NHS Trust (EEAST) will be examined. We will examine PCRs from a 1 to 2 days period in January and a 1to 2 days period in July, to allow for seasonal variation. This is likely to give data on ∼700 calls from EEAST and 2000 calls from SWASFT (based on 2014 ambulance service data provided for the purposes of this research).

### Methods

SWASFT PCRs from calls to patients aged 65 years and over within the geographical area covered by the SWASFT West Division will be accessed via a bespoke query run on the Trust's electronic care system and the anonymised data sent to the research team. In EEAST, PCRs will be copied, anonymised by EEAST staff, and sent to the research team.

The researcher will examine both sets of records using a standard template and record the data using a coding structure. The study team will determine the template and coding in advance by obtaining a pilot sample from a different time period of 40 PCRs which will be scrutinised for relevant data. Data from the pilot will not be analysed for the study. Development of the template will be informed by recent work on the use of EMS by older people with dementia which provides a benchmark for others looking at ambulance service data.^18^ The template will guide the researcher in obtaining information on the time of the call, the length of time the call took, the reason for the call, whether dementia and any other multimorbidities were noted, whether the patient was in their own home or a care home, and if they were living alone, whether the patient was conveyed to hospital and, if not, any further referrals that were made. A sample of 40 PCRs will be examined by additional members of the research team and subject to assessment of inter-rater reliability using the κ statistic. A coding structure will be used to quantify the data.

### Analysis

From these cleaned data, it will be possible to report as percentages with 95% CIs:

- How often dementia is recorded;
- How often another multimorbidity is recorded;
- How often dementia and another multimorbidity are recorded for the same call;
- Whether the person was in their own home or in a care home, and if they were living alone;
- The proportion of patients with multimorbidities including dementia that were transported to hospital, in comparison to control calls where multimorbidities not including dementia were recorded;
- How long the call lasted.

Descriptive statistics appropriate for continuous data will be used to compare how long call-outs to people with multimorbidities including dementia last, in comparison to control call-outs where dementia is not recorded. Reasons for transporting patients to hospital will also be categorised and described.

### Phase 2: observational case studies

This work will use qualitative methods, a combination of observation, interview and documentation analysis, to study the factors influencing the decision-making process during call-outs to older people with multimorbidities including dementia. A phenomenological approach will be adopted to study the subjective experiences of paramedics and the impact of factors such as organisation, resources and family wishes on their decisions regarding conveyance to hospital. This will be in the form of up to 20 observational case studies on older people with multimorbidities including dementia who are the subject of a 999 call to the ambulance service.

## Methods

### Sampling

It is important to include both rural and urban areas of SWASFT in the case studies, to take into account variation in the availability of services. Therefore, four paramedics will be recruited: two from rural areas and two from urban areas. Five case studies will be obtained with each paramedic to make a total of 20.

### Selection, recruitment and consent

In order for a case to be studied, an eligible patient needs to be attended to by a participating paramedic.

Patients will be eligible for a case study if they:

- (Or someone on their behalf) has called an emergency ambulance;
- Are aged 65 years or older;
- Have an established diagnosis of dementia and at least one multimorbidity;
- Consent (or have a consultee consent) to observation of the call and analysis of the call records.

Paramedics will be eligible to participate if they:

- Agree to be observed, recorded and interviewed;
- Consent to participate.

Establishing diagnosis of dementia:

1. Documentary evidence at the scene that the patient has a dementia diagnosis. This may be in the form of paperwork or a care plan left by visiting care staff or one of the recognised ways of communicating information in the ambulance service such as the ‘message in a bottle’ scheme.
2. Verbal confirmation from the patient and/or the carer that they have been diagnosed with dementia by a general practitioner or hospital doctor.
3. Patients already known to the ambulance service from previous calls, and identified as a person with dementia on the call record.

If no evidence of any of the above is available, it will be assumed that the patient does not have an established diagnosis of dementia, and they will not be eligible for inclusion in the study.

A qualitative researcher (QR) will be employed to shadow ambulance paramedics and attend calls to people aged 65 years or more who are not known to have a life-threatening or critical condition. The practice of ‘Third Crewing’ (when an individual works with crews on ambulances to gain experience) is common in the ambulance service, and there are SWASFT procedures in place to enable this. On the basis of analysis of existing call data, we expect that on average a paramedic will attend one eligible patient in each 12-hour shift. The QR will therefore cover at least 20×12 hours shifts (five shifts which each of the four paramedics) during the 6-month data collection period. The shifts will be at different time points and on different days to take account of variation in the availability of services in and out of hours. Of the five shifts, three will be day shifts (one of these at a weekend) and two will be night shifts (one at a weekend). During each shift, they will be on site at the paramedic's base station and will attend calls with the paramedic, but remain in the vehicle on arrival. Once on scene and after initial assessment, the participating paramedic will screen the patient for eligibility.

If the patient is eligible, the paramedic will provide basic study details using scripted information and ask if the patient is happy to speak to the QR. If they are eligible and they have capacity, the QR will approach the patient, provide further study information and take written informed consent. If they are eligible and do not have capacity but have a personal consultee (spouse or family member) present, the paramedic will provide basic study information and ask if the consultee is happy to speak with the QR who will provide detailed study information and ask for a written response. If either the patient or consultee expresses a wish to not take part, the QR will leave the scene and rejoin the paramedic after they have completed the call. Patients or consultees will have three consent options: no participation; passive participation or active participation. For passive participation, the QR will collect all data from observation, paramedic interview and document analysis. For active participation, the QR will contact the patient or consultee to undertake an interview at a later date. This method of approaching the patient and carer, and seeking consent, has been approved by our patient and public partners.

### Data collection

If a patient or consultee consents, the QR will observe and record all assessments and interactions to collect data. There will be four elements to the data collection: observation, paramedic interview, documentation analysis and retrospective patient interview.

1. *Observation*: the QR will document assessments, input from family members or others present on the scene, actions taken by the paramedic through the use of field notes taken immediately, and expanded notes made on reflection. The researcher will note what is observed and what thoughts this generates. These reflections help the researcher note their own bias or early analysis.
2. *Paramedic interview*: the QR may ask the paramedic for clarification on decisions made and the rationale for these both during and after the call. This will include a description of what happened and what they were thinking about during the clinical encounter.
3. *Document analysis*: the QR will take a copy of the PCR and any referral documents that the paramedic completes during the course of the call and any subsequent handover.
4. *Retrospective patient or carer interview*: if the patient or carer consents to the follow-up, the QR will contact them 1 week after the incident to ask them about their experience of the 999 call-out and any further healthcare contacts or interventions. These interviews will be conducted retrospectively to reflect on the advice received from our patient and public involvement (PPI) partners during the development of the protocol.

The observational data will be in the form of field notes, which will be transcribed to enable sharing with co-researchers. Interviews will be audio-recorded, with consent, and transcribed. Transcription will be conducted by an independent transcriber who will de-identify the data. Data will then be coded by the QR and another member of the research team.

### Data analysis

#### First-level analysis

Each of the four data sources will first be analysed as a stand-alone data set. A well-established iterative process of data reduction, constant comparison, organisation and understanding through thematic analysis will be used to analyse each data source.^19^

1. Familiarisation with the data: reading and rereading transcripts;
2. Generating initial codes: noting codes of interesting and pertinent ideas;
3. Searching for themes: systematically organising these recurrent ideas with extracts of text;
4. Reviewing themes: checking themes are meaningful and relate to the text;
5. Defining and naming themes: summarising the narrative with clear definitions;
6. Producing the report: using extracts of data to exemplify the themes;

The themes derived from the analysis will be grounded in these data, without predefined theory or assumptions.

Each of these data sets will be analysed by the primary researcher with a sample checked by a second researcher for plausibility and validity. Consensus of themes will need to be reached.

#### Second-level analysis

Each data source will be triangulated against the other to test for similarity and differences, where contradictions and consistencies in the data occur. Deviant cases will be actively sought. The phenomenological approach means that analysis will primarily explore the experiences of the paramedics, and focus on the accounts they give of their decision-making and contextual factors that influence these decisions. Similar to the first-level analysis, the triangulation and subsequent themes will be checked by a second researcher and consensus will need to be reached. It is not anticipated that the multiple data sources will provide an exhaustive account and an ultimate truth, but will illuminate a mechanism of decision-making and illustrate the influences on this process. It is planned that a model of types of decision-making will be built from these findings and that case studies will also contribute to phase 3 of the project with example vignettes to present back to paramedics.

### Phase 3: needs analysis

With the focus on prehospital and urgent care, a needs analysis will be conducted to identify the components required in an intervention to reduce avoidable A&E attendances and emergency hospital admissions for older people with multimorbidities including dementia.

#### Methods and analysis

Participants will be SWASFT paramedics invited through the ambulance service and who consent to attend a nominal group meeting. There will be three meetings that will be held as part of the existing SWASFT training or research events. These will be convenience samples and participants will give informed consent. Participation will be voluntary but it will be important to engage a wide range of paramedics from different geographical areas and roles. Records will be made of participant demographic and role information. Where gaps in paramedic role or location occur in the samples, purposive sampling will be adopted.

The purpose of the groups will be to consider what resources would be appropriate and effective in supporting alternatives to A&E attendance and hospital admission for older people with multimorbidities including dementia. By presenting vignettes derived from the phase 2 data collection, the paramedics will be asked to consider what resources would enable those who deliver emergency, urgent and out of hours care to manage such patients at home or in a community setting, rather than conveying to A&E by ambulance with a high likelihood of consequential acute hospital admission. They will also be asked to prioritise these resources using nominal group technique. In this technique, participants respond to the vignette by silent reflection, individually brainstorming and then prioritising their own ideas on which resources would be appropriate and potentially effective. Once this is done, the participants share their ideas with the group in a round-robin format. These ideas are noted by the researcher using flip charts, are visible to all and can be openly discussed, developed, clarified and challenged by the group. Where there is repetition of ideas, this will be noted. After discussion, the group members anonymously vote on the prioritisation of the ideas listed by the group members. They have five votes each which rank the level of priority (1—least important, 5—most important). This prioritisation or ‘voting’ allows the researcher to rank the ideas with the most votes and illustrate the most popular ideas.^20^ Additionally, notes will be made about the discussions arising to give additional context to the prioritisation process and contribute to reporting.

This technique is useful to engage all members of the group, where some might not feel able to contribute and where some people in the room may occupy powerful roles. It includes everyone's ideas and results in consensus decision-making by the experts in the room. It is also a time-efficient way of engaging experts in influencing research and practice.^21^

## Ethics and dissemination

Patients and public have been fully engaged in identifying the research topic and in the preparation of this protocol. This study is being partnered by the Alzheimer's Society for Patient and Public Involvement. PPI partners were consulted for the drafting of study documents to ensure that patient information leaflets and consent forms reflect the agreed consent process. An experienced patient and public representative is a member of the Study Management Group and a further two PPI members are members of the steering committee.

A dissemination strategy will be implemented that includes electronic dissemination of the study outputs to ambulance services in the UK, as well as to acute trusts, mental health trusts, dementia charities and through a publicly accessible website. We will also provide feedback to all stakeholder groups and present our findings at relevant conferences and at national ambulance, dementia and emergency care meetings.

Findings will be published in appropriate journals, presented at conferences and circulated in newsletters. We will pay particular attention to dissemination to the public, dementia organisations and ambulance services. There will be important implications for further and ongoing research and we will ensure that we make our findings freely available for future adaptation and use.

## Discussion

To the best of our knowledge, HOMEWARD will be the first study to quantify the amount of time ambulance staff spend on the scene when called to people with dementia and other multimorbidities. Furthermore, the use of non-participant observation is a novel approach to examining the factors influencing the decision to convey to hospital, or not, in complex situations involving patients with multiple health problems. It is anticipated that HOMEWARD will generate findings that inform the development of service-level interventions to reduce avoidable hospital admissions for older people.
